# Fibrosis-4 (FIB-4) index as a predictor for mechanical ventilation and 30-day mortality across COVID-19 variants

**DOI:** 10.1017/cts.2023.594

**Published:** 2023-07-17

**Authors:** Priyanka Parajuli, Roy Sabo, Rasha Alsaadawi, Amanda Robinson, Evan French, Richard K. Sterling

**Affiliations:** 1 Department of Internal Medicine, Virginia Commonwealth University, Richmond, VA, USA; 2 C. Kenneth and Dianne Wright Center for Clinical and Translational Research, Virginia Commonwealth University, Richmond, VA, USA; 3 Department of Biostatistics, Virginia Commonwealth University, Richmond, VA, USA; 4 Division of Gastroenterology, Hepatology, and Nutrition, Virginia Commonwealth University, Richmond, VA, USA; 5 Division of Infectious Disease, Virginia Commonwealth University, Richmond, VA, USA

**Keywords:** COVID-19, mechanical ventilation, FIB-4

## Abstract

**Background:**

The Fibrosis-4 (FIB-4) index, a simple index that includes age, liver enzymes, and platelet count has been studied as a tool to identify patients at a risk of requiring mechanical ventilation due to its high negative predictive value. It is unknown if FIB-4 remains useful to predict the severity of respiratory disease requiring mechanical ventilation amongst new Coronavirus disease 2019 (COVID-19) variants and whether a relationship also exists between FIB-4 and 30-day mortality. The main objective was to determine if FIB-4 can predict mechanical ventilation requirements and 30-day mortality from COVID-19 across variants including Alpha, Delta, and Omicron.

**Methods:**

This was a population-based, retrospective cohort analysis of 232,364 hospitalized patients in the National COVID-19 Cohort Collaborative between the age of 18–90 who tested positive for COVID-19 between April 27, 2020 and June 25, 2022. The primary outcome was association between FIB-4 and need for mechanical ventilation. Secondary measures included the association of FIB-4 with 30-day mortality.

**Results:**

A FIB-4 > 2.67 had 1.8 times higher odds of requiring mechanical ventilation across all variants of COVID-19 (OR 1.81; 95% CI: [1.76, 1.86]). The area under the ROC curve showed high diagnostic accuracy with values ranging between 0.79 (Omicron wave) and 0.97 (delta wave). Increased FIB-4 was associated with 30-day mortality across the variates.

**Conclusion:**

The FIB-4 was consistently associated with both increased utilization of mechanical ventilation and 30-day mortality among COVID-19 patients across all waves in both adjusted and unadjusted models. This provides a simple tool for risk-stratification for front-line health care professionals.

## Introduction

The Coronavirus disease 2019 (COVID-19) pandemic, caused by severe acute respiratory syndrome coronavirus (SARS-CoV-2), has been associated with high prevalence of respiratory failure requiring mechanical ventilation (MV) [1]. The continuous evolution of the virus has led to new variants of concern (VOC) that vary in transmissibility, severity or change in clinical presentation, and response to vaccinations [2,3]. As of December 2022, the subsequent Omicron variant is now the dominant variant and makes up greater than 98% of the detected viral sequences. Recent studies of the Omicron variant have shown reduced odds of hospitalization with Omicron vs. the prior Delta variant [4–6]. The Fibrosis-4 (FIB-4) index for liver fibrosis, developed to estimate fibrosis in chronic liver disease using age, aspartate aminotransferase (AST) alanine aminotransferase (ALT) levels, and platelet counts, has been associated with need for MV in COVID-19 [1,7,8]. A study by Li *et al*. [9] showed that a FIB-4 > 2.67 (the threshold to detect advanced fibrosis in nonalcoholic fatty liver disease [NAFLD]) had higher mortality rates and was attributed to a positive correlation between FIB-4 and the level of SARS-CoV-2 viral load and inflammatory cytokines. It also studied elevated plasma levels of the SARS-CoV-2 RNA as an association with the FIB-4 level. Temporal analysis later in the hospitalization showed improvement in FIB-4 levels amongst survivors of COVID-19. Younossi *et al.* [10] studied patients with NAFLD and determined that the FIB-4 score was an independent predictor of mortality from COVID-19 by multiple regression analysis. A recent study by Sterling *et al.* has demonstrated the utility of using FIB-4 as a tool to identify patients more likely to require MV due to its high negative predictive value (NPV), identifying an optimal FIB-4 cutoff point of > 3.04 to predict need for MV [1,11]. These studies have shown the utility of FIB-4 as risk-stratification tool for patients diagnosed with COVID-19. Other complex models like the 4C mortality score, BAS^2^IC score, and the red cell distribution width (RDW) have been studied to predict mortality from COVID-19. However, these scores have been challenging to use in clinical practice due to the use of non-routine laboratory values and the requirement of subjective symptoms in the calculations [12–14]. We have previously shown that FIB-4 is superior to RDW in predicting MV in COVID-19, as RDW did not show an association of increased RDW on MV [1]. Due to the evolving nature of SARS-CoV-2, it is useful to reassess the validity of FIB-4 as a predictive tool. We hypothesize that a FIB-4 level>3.04, a value identified during the initial variant, can predict respiratory failure requiring MV with the COVID-19 variants Alpha, Delta, and Omicron and their associated mortality in hospitalized patients.

## Methods

### Patient Characteristics

This was a population-based retrospective cohort analysis of COVID-19 patients in the United States using the National COVID-19 Cohort Collaborative (N3C) [15,16]. We identified 232,364 patients who tested positive for COVID-19 between April 27, 2020 and June 25, 2022 with sufficient data to calculate BMI and FIB-4. Of those, 47,919 were hospitalized during the initial COVID-19 wave, 12,207 during the Alpha wave, 38,187 during the Delta wave, 34,871 during the Omicron-initial wave, and 6,915 during the Omicron-subsequent wave. COVID-19 waves (supplemental Table 1; Table S1) were defined using data ranges, and waves were not contiguous to allow time for the prevalent variant to shift. Analyses were restricted to patients between the ages of 18 and 90. Patients were further restricted to those with AST, ALT, and platelet measurements within 24 hours of hospitalization. FIB-4 was calculated as age (year) x AST (U/L) / [platelet count (10^9^/L) x √ALT (U/L)] [5]. Continuous values were required to fall in the following ranges (and were excluded, otherwise): AST: 0-1000 U/L, ALT: 0-1000 U/L, platelets: 20–4000 10^9^/L, FIB-4: 0.2–20. Individual patient demographic and clinical characteristics were summarized by COVID-19 wave. Frequencies and percentages were reported for categorical variables, while means and standard deviations were reported for continuous variables. Figure S1 summarizes the sample size starting from the total number of COVID-19-positive patients, then those between age 18–90, and those with FIB-4 components (ALT, AST, PLT) within valid ranges. Patients with an invalid death date or sex were also excluded.

**Table 1.** Odds ratios of mechanical ventilation using simple vs multiple logistic regression models for Alpha, delta, omicron-initial, and omicron-subsequent waves

		Alpha		Delta		Omicron-initial		Omicron-subsequent	
Wave		SLR	MLR	SLR	MLR	SLR	MLR	SLR	MLR
Variables		OR (95% CI)	OR (95% CI)	OR (95% CI)	OR (95% CI)	OR (95% CI)	OR (95% CI)	OR (95% CI)	OR (95% CI)
Intercept		–	0.01 (0.01, 0.01)	–	-–	0.01 (0.01, 0.01)	0.02 (0.01, 0.02)	–	0.02 (0.02, 0.03)
Female		0.79 (0.70, 0.90)	0.96 (0.82, 1.12)	0.75 (0.71, 0.80)	0.67 (0.65, 0.69)	0.74 (0.72, 0.77)	0.69 (0.64, 0.76)	0.06 (0.05, 0.07)	0.73 (0.59, 0.91)
Age		1.04 (1.00, 1.08)	–	0.98 (0.96, 1.00)	1.00 (0.99, 1.01)	–	–	0.93 (0.89, 0.98)	–
Race^1^	Native American	1.48 (0.52, 4.23)	1.06 (0.32, 3.48)	2.20 (1.46, 3.31)	2.13 (1.77, 2.58)	1.52 (1.21, 1.90)	1.78 (0.99, 3.20)	0.90 (0.12, 6.77)	0.55 (0.06, 5.28)
	Asian	1.54 (1.10, 2.16)	1.51 (1.01, 2.25)	1.73 (1.36, 2.21)	1.52 (1.42, 1.64)	1.51 (1.39, 1.65)	1.39 (1.07, 1.82	1.08 (0.62, 1.87)	1.16 (0.63, 2.12)
	Black	0.83 (0.71, 0.98)	0.73 (0.60, 0.89)	0.96 (0.88, 1.05)	1.09 (1.05, 1.13)	1.01 (0.97, 1.06)	0.99 (0.89, 1.10)	0.89 (0.66, 1.20)	0.91 (0.66, 1.25)
	Native Hawaiian	1.59 (0.36, 7.02)	2.17 (0.43, 10.83)	0.73 (0.26, 2.05)	1.34 (0.96, 1.88)	1.25 (0.85, 1.84)	1.58 (0.54, 4.58)	0 (0, inf)	0 (0, inf)
	Hispanic	1.02 (0.83, 1.24)	0.79 (0.62, 1.02)	1.18 (1.06, 1.32)	1.37 (1.32, 1.42)	1.28 (1.22, 1.34)	1.18 (1.02, 1.36)	0.81 (0.53, 1.24)	1.00 (0.64, 1.57)
	Other	0.80 (0.35, 1.85)	1.09 (0.42, 2.82)	0.78 (0.52, 1.16)	0.94 (0.79, 1.10)	1.11 (0.92, 1.34)	1.54 (1.02, 2.32)	1.47 (0.45, 4.83)	1.59 (0.42, 5.93)
	Unknown	0.89 (0.67, 1.18)	0.76 (0.54, 1.07)	1.14 (0.99, 1.32)	1.21 (1.14, 1.28)	1.21 (1.13, 1.29)	1.04 (0.86, 1.27)	1.14 (0.77, 1.69)	1.37 (0.90, 2.08)
Cardiac disease		1.19 (1.03, 1.37)	0.91 (0.77, 1.09)	0.82 (0.76, 0.88)	0.86 (0.84, 0.89)	0.71 (0.68, 0.73)	0.73 (0.67, 0.80)	0.87 (0.71, 1.07)	0.77 (0.61, 0.97)
Diabetes		1.63 (1.43, 1.87)	1.26 (1.06, 1.49)	1.46 (1.37, 1.55)	1.56 (1.52, 1.60)	1.28 (1.24, 1.33)	1.03 (0.94, 1.13)	1.10 (0.89, 1.36)	1.00 (0.79, 1.28)
Liver disease		2.56 (2.16, 3.03)	2.01 (1.63, 2.49)	1.83 (1.68, 1.99)	1.89 (1.82, 1.96)	1.54 (1.47, 1.61)	1.46 (1.31, 1.62)	1.39 (1.08, 1.80)	1.11 (0.83, 1.49)
Respiratory disease		1.11 (0.93, 1.33)	1.04 (0.83, 1.30)	0.91 (0.84, 0.99)	0.85 (0.82, 0.88)	0.90 (0.86, 0.95)	0.90 (0.81, 1.00)	0.85 (0.67, 1.09)	0.92 (0.70, 1.22)
BMI≥30		1.66 (1.45, 1.90)	1.37 (1.16, 1.61)	2.09 (1.96, 2.23)	1.74 (1.60, 1.88)	1.59 (1.48, 1.71)	1.43 (1.31, 1.56)	1.03 (0.84, 1.27)	1.11 (0.88, 1.40)
Days in hospital		1.10 (1.09, 1.10)	1.09 (1.09, 1.10)	1.11 (1.10, 1.11)	1.10 (1.09, 1.10)	1.08 (1.08, 1.08)	1.07 (1.07, 1.08)	1.07 (1.06, 1.08)	1.07 (1.06, 1.08)
Treatment		2.45 (2.14, 2.81)	2.09 (1.77, 2.46)	3.08 (2.87, 3.30)	2.28 (2.10, 2.48)	2.35 (2.18, 2.53)	1.76 (1.62, 1.92)	0.95 (0.78, 1.17)	0.83 (0.66, 1.04)
AST*		1.04 (1.03, 1.05)	–	1.05 (1.04, 1.05)	–	1.04 (1.03, 1.04)	–	1.05 (1.04, 1.06)	–
ALT*		1.02 (1.01, 1.03)	–	1.03 (1.02, 1.03)	–	1.03 (1.03, 1.03)	–	1.04 (1.03, 1.05)	–
PLT*		1.00 (0.99, 1.00)	–	0.99 (0.99, 1.00)	–	0.99 (0.99, 0.99)	–	0.99 (0.98, 1.00)	–
FIB-4^		1.25 (1.24, 1.26)	1.22 (1.21, 1.23)	1.25 (1.22, 1.28)	1.21 (1.18, 1.24)	1.22 (1.21, 1.24)	1.20 (1.19, 1.21)	1.20 (1.19, 1.22)	1.19 (1.18, 1.20)
FIB-4 > 2.67		1.84 (1.61, 2.10)	–	1.84 (1.73, 1.96)	–	1.89 (1.75, 2.03)	–	1.68 (1.37, 2.06)	–
FIB-4 > 3.04		1.97 (1.72, 2.26)	–	1.86 (1.74, 1.98)	–	1.87 (1.74, 2.02)	–	1.71 (1.39, 2.11)	–
FIB-4 > 3.25		1.90 (1.66, 2.18)	–	1.83 (1.72, 1.96)	–	1.90 (1.76, 2.05)	–	1.74 (1.40, 2.15)	–

AST = alanine aminotransferase; BMI = body mass index; FIB-4 = fibrosis-4 (FIB-4); PLT = platelets; MLR = multiple logistic regression; SLR = simple logistic regression.

* Odds ratios for 10-unit difference.

^ Odds ratios for one unit increase in FIB-4.

1 The reference group is White.

### Statistical Analysis

Simple logistic regression (SLR) models for odds ratio (OR) were utilized by specific COVID-19 wave to investigate potential bivariate associations between MV use, 30-day mortality, and the following patient characteristics: age; AST; ALT; platelets; sex; race/ethnicity; diabetes; comorbid cardiac, liver, and respiratory disease; obesity; days in the hospital; admission to ICU within 30 days of COVID-19 diagnosis; COVID-19 treatment within 30 days of diagnosis; FIB-4; and indicators of whether FIB-4 > 2.67 (the threshold for advanced fibrosis in NAFLD) [17], FIB-4 > 3.04 (the threshold identified in COVID-19) [1], or FIB-4 > 3.25 (the threshold for advanced fibrosis in chronic viral hepatitis) [5]. Multiple logistic regression (MLR) models were fit by COVID-19 wave to investigate associations between MV use, 30-day mortality, and FIB-4, adjusting for sex; race/ethnicity; diabetes; comorbid cardiac, liver, and respiratory disease; obesity; admission to ICU within 30 days of COVID-19 diagnosis; and COVID-19 treatment within 30 days of diagnosis. We defined the 30-day outcome as binary (mortality within 30 days or not): a patient was assumed to be alive unless their death was indicated in the data. All covariates were included in the MLR models, reproducing the measures and approach used in our previous work [1].

To help reduce bias, internal validation was used by splitting the data into training (70%) and testing (30%) subsets. The training set was used to fit MLR models between the continuous FIB-4 covariate and the use of MV for COVID-19 variant wave; models were also fit using each one of the categorical FIB-4 covariates (FIB-4 > 2.67, FIB-4 > 3.04, FIB-4 > 3.25), and are provided as supplementary material. From the testing subsets, we estimate area under the receiver operating characteristic (AUROC) curve, sensitivity, specificity, positive predictive value (PPV), negative predictive value (NPV), and AUROC for each COVID-19 variant wave is reported. Note that while the categorical FIB-4 measures are used directly to estimate agreement statistics, for the continuous FIB-4 model, we used Youden’s Index to determine the probabilistic cutoff for classifying the resulting predicted probabilities as “high” or “low” likelihood of MV use, which is then used to estimate agreement.

SLR was utilized to investigate bivariate associations between death within 30 days of hospitalization and each of the following patient characteristics: age; AST; ALT; platelets; sex; race/ethnicity; diabetes; comorbid cardiac, liver, and respiratory disease; obesity; days in the hospital; admission to ICU within 30 days of COVID-19 diagnosis; COVID-19 treatment within 30 days of diagnosis; and FIB-4. MLR models for 30-day mortality were also adjusted for sex; race/ethnicity; diabetes; comorbid cardiac, liver, and respiratory disease; obesity; days in hospital; COVID-19 treatment within 30 days of diagnosis; and FIB-4.

## Results

### Demographics and Clinical Characteristics

The demographic and clinical characteristics of patients including percentages of comorbid diseases by COVID-19 wave are summarized in Table S2. Across waves, mean patient age ranged from 56 to 62 years old, while mean FIB-4 ranged from 2.58 to 2.94. The rate of MV varied across COVID-19 waves ranging from 10% during the initial wave, 8% during Alpha, 12% during Delta, 9% during Omicron-initial wave, and 6% during the Omicron-subsequent wave. During the initial variant, about 9% of patients died in the hospital, 11% during the Delta wave, and 4% during the subsequent Omicron wave.

### Predictors of Mechanical Ventilation

Table 1 summarizes bivariate and multivariable results from logistic regression models by COVID-19 wave for MV. Comorbid liver disease or diabetes had higher odds of MV compared to those without across all COVID-19 waves in the unadjusted and adjusted models, and this effect was consistent within waves. Table 1 and Table S3 show the OR of various comorbid diseases with MV across all waves. Odds of MV increased with length of hospital stay across all COVID-19 waves. This effect persisted for each COVID-19 wave even after adjusting for other factors (OR 5.41 [5.13, 5.71]). Patients who received COVID-19 treatment within 30 days of diagnosis had higher odds of MV use than those who did not receive treatment across all waves. The OR of MV use was higher for those who had treatment in all waves.

In the unadjusted model, a FIB-4 > 2.67 had an OR [95% CI] ranging between 1.68 [1.37, 2.06] (subsequent Omicron) to 1.89 [1.75, 2.03] (Omicron). A FIB-4 > 3.04 had an OR ranging from 1.71 [1.39, 2.11] (subsequent Omicron) to 1.97 [1.72, 2.26] (Alpha). A FIB-4 > 3.25 had an OR ranging from 1.74 [1.40, 2.15] (subsequent Omicron) to 1.90 [1.76, 2.05] (Omicron and Alpha). Figure S2 and Figure S3 show ORs and 95% confidence intervals for FIB-4 as a continuous variable and for FIB-4 > 3.04 by COVID-19 variant wave and model respectively.

The unadjusted OR of MV for one unit increase in FIB-4 across all waves was 1.12 [1.11, 1.12]. OR estimates for FIB-4 were similar across COVID-19 waves. The adjusted OR of MV use for one unit increase in the FIB-4 index across all waves was 1.09 [1.09, 1.10] and was similar among waves. For the components of FIB-4, AST was significantly associated with MV at a univariable level across COVID-19 waves. The OR for MV use for a 10-unit increase in AST was 1.04 [1.04, 1.04] across all COVID-19 waves. ALT was significantly associated with MV at a univariable level across COVID-19 waves. The OR of MV use for a 10-unit increase in ALT was 1.03 [1.03, 1.03] across all COVID-19 waves. Platelet level was not significantly associated with MV at a univariable level for any except the Omicron-initial wave. The OR of MV for a 10-unit increase in platelets was 0.99 [0.99, 1.00] across all COVID-19 waves. The OR of MV for a 10-unit increase in age is significantly higher than one in the initial and Alpha COVID-19 waves, significantly lower than one in Omicron-subsequent wave, and not significantly different in the Delta and Omicron-initial waves.

**Figure 1.**
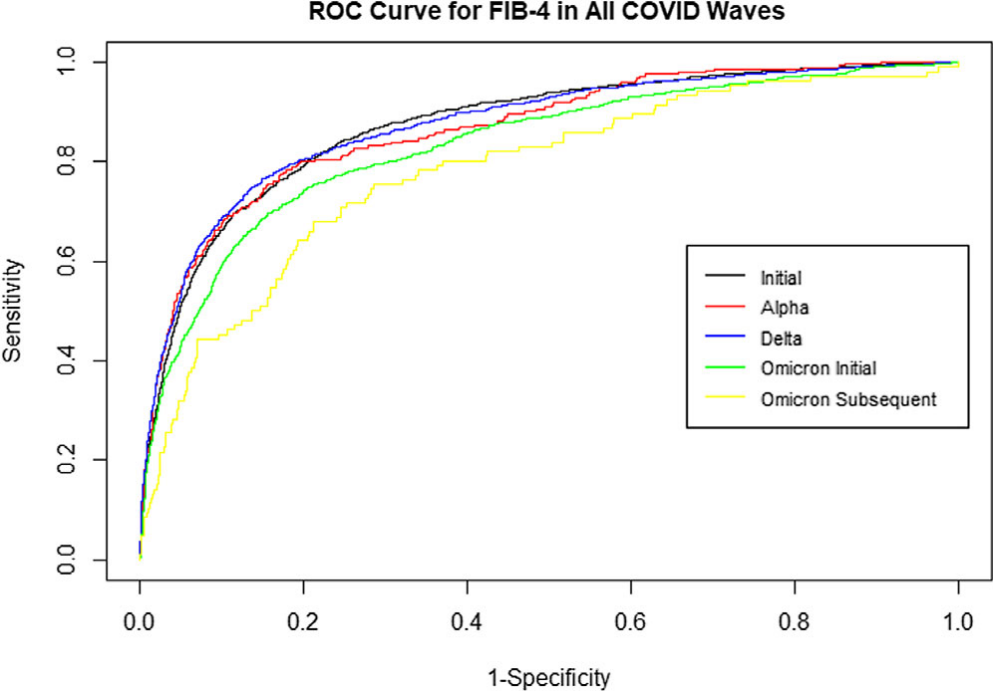
Receiver operating characteristic (ROC) curve for fibrosis-4 (FIB-4) across all COVID-19 variants.

Using cross-validation and MLR models for each COVID-19 wave, we calculated the AUROC between the continuous FIB-4 covariate, FIB-4 > 2.67, FIB-4 > 3.04, and FIB-4 > 3.25, and the use of MV per COVID-19 variant wave. For each categorical FIB-4 indicator, the predictability of MV by FIB-4 was calculated using the Youden Index, the sensitivity, specificity, PPV, NPV, and AUROC for each COVID-19 wave (Table 2). The AUROC curves between the continuous FIB-4 covariate and the use of MV per COVID-19 variant wave also ranged from 0.78–0.87 across different waves (Figures S4–S8). It showed a high sensitivity ranging from 0.78 to 0.80 for the initial variant, 0.74 for Alpha, 0.76–0.78 for Delta, 0.70–0.71 for Omicron, and 0.67–0.71 for the subsequent Omicron variant. The specificity ranged from 0.83 to 0.84 across all variants. The NPV ranged from 0.97 to 0.98 across variants. The PPV, however, was low (0.33–0.34). Fig. 1 shows the ROC curve plots for each COVID-19 wave for FIB-4 as a continuous variable.

**Table 2.** Predictability of mechanical ventilation by FIB-4

		Initial/untyped	Alpha	Delta	Omicron-initial	Omicron-subsequent	r
FIB-4	AUROC	0.87	0.87	0.87	0.83	0.79	
FIB-4 > 2.67	AUROC	0.87	0.86	0.87	0.82	0.78	
	Sensitivity	0.78	0.74	0.76	0.71	0.71	
	Specificity	0.81	0.86	0.85	0.81	0.75	
	PPV	0.31	0.32	0.40	0.26	0.14	
	NPV	0.97	0.97	0.96	0.97	0.98	
FIB-4 > 3.04	AUROC	0.87	0.87	0.87	0.82	0.78	
	Sensitivity	0.80	0.74	0.78	0.70	0.69	
	Specificity	0.79	0.87	0.83	0.81	0.76	
	PPV	0.30	0.33	0.37	0.27	0.15	
	NPV	0.97	0.97	0.97	0.97	0.98	
FIB-4 > 3.25	AUROC	0.87	0.87	0.87	0.82	0.78	
	Sensitivity	0.80	0.74	0.77	0.70	0.67	
	Specificity	0.80	0.87	0.84	0.81	0.78	
	PPV	0.30	0.33	0.39	0.26	0.16	
	NPV	0.97	0.97	0.96	0.97	0.98	

AUROC = area under the receiver operating characteristic; FIB-4 = fibrosis-4; NPV = negative predictive value; PPV = positive predictive value.

### Association of FIB-4 and 30-day Mortality

**Figure 2.**
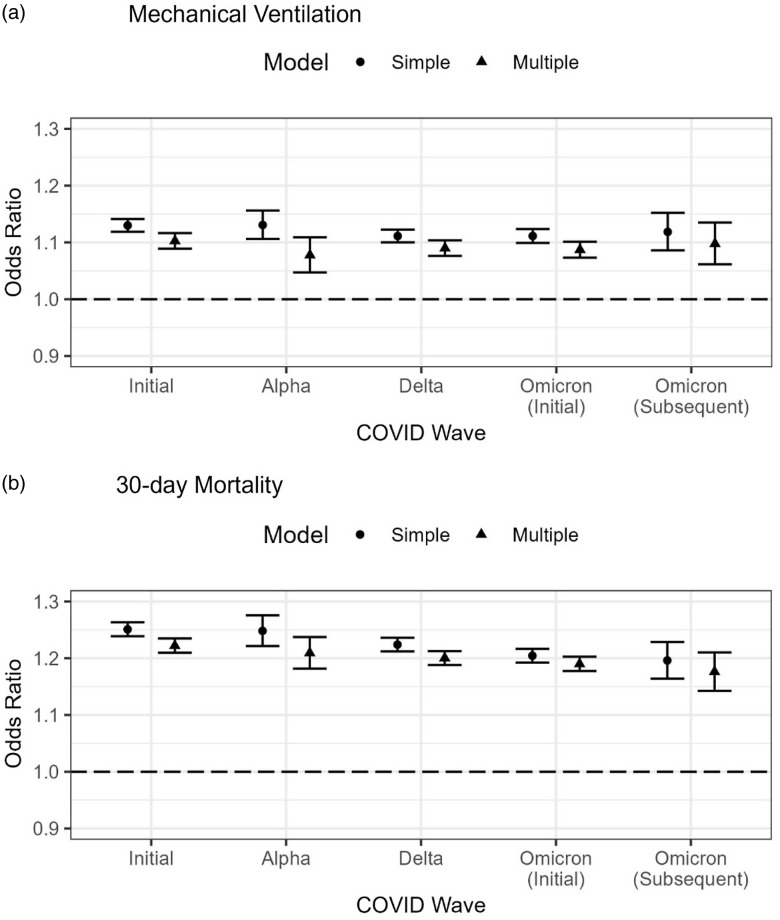
Odds ratios using simple vs. multiple logistic regression modeling for mechanical ventilation (a) and 30-day mortality (b) for a one unit change in continuous FIB-4 by COVID-19 variant.

We identified 25,250 patient deaths within 30 days of hospitalization, 4,948 during the initial wave, 858 during the Alpha wave, 4,697 during the Delta wave, 3,583 during the Omicron-initial wave, and 374 during the Omicron-subsequent wave. Table 3 and Fig. 2 summarize ORs for 30-day mortality using simple logistic regression (SR) vs. multiple logistic regression (MR) for the initial, alpha, delta, omicron-initial and omicron-subsequent Waves. The adjusted OR [95% CI] by MR for FIB-4 across all waves was 1.21 [1.20, 1.21] and was similar for each wave. Fig. 2 shows OR and 95% confidence intervals for MV and 30-day mortality for FIB-4 by COVID-19 variant. All the components of FIB-4 were significantly associated with 30-day mortality without adjusting for other factors within waves. The unadjusted OR for a one-year increase in age was 1.05 [1.05, 1.05] across all waves. Mortality risk increased across all waves with AST (per 10 U/L increase) with an OR of 1.04 [1.04, 1.04] and ALT (per 100 U/L increase) with an OR of 1.01 [1.01, 1.02], not adjusting for other factors. Mortality risk decreased as platelets (per 10 10^9^/L increase) increased with an OR of 0.98 [0.98, 0.98] across all waves and similar effects within each wave. COVID-19 treatment within 30 days of diagnosis had a significantly higher likelihood of death compared to those who did not receive treatment in the unadjusted model with an OR of 1.26 [1.23, 1.29] across all waves. This effect persisted within all waves except the Alpha wave, where no difference in likelihood was observed. After adjusting for other factors, the OR with treatment decreased in the initial, delta, and omicron waves, and showed no difference in the Alpha and subsequent Omicron waves.

**Table 3.** Odds ratios for 30-day mortality using simple vs. multiple logistic regression models for the initial, Alpha, delta, omicron-initial, and omicron-subsequent waves

		Initial		Alpha		Delta		Omicron initial		Omicron subsequent	
Wave		SR*	MR^†^	SLR	MLR	SR	MR	SR	MR	SR	MR
Variables		OR (95% CI)	OR (95% CI)	OR (95% CI)	OR (95% CI)	OR (95% CI)	OR (95% CI)	OR (95% CI)	OR (95% CI)	OR (95% CI)	OR (95% CI)
Male		1.36 (1.28, 1.44)	1.16 (1.09, 1.24)	1.31 (1.14, 1.51)	1.15 (1.00, 1.34)	1.25 (1.18, 1.33)	1.14 (1.07, 1.22)	1.39 (1.30, 1.49)	1.21 (1.12, 1.30)	1.48 (1.20, 1.83)	1.26 (1.01, 1.57)
Age		1.06 (1.06, 1.06)	–	1.05 (1.05, 1.06)	–	1.04 (1.04, 1.04)	–	1.04 (1.04, 1.05)	–	1.04 (1.03, 1.04)	–
Race	Native American	1.37 (0.91, 2.00)	1.23 (0.79, 1.85)	1.47 (0.44, 3.75)	1.48 (0.42, 3.95)	0.92 (0.53, 1.49)	0.71 (0.39, 1.21)	1.74 (1.03, 2.81)	1.51 (0.86, 2.53)	0.87 (0.05, 4.23)	0.63 (0.03, 3.69)
	Asian	0.71 (0.59, 0.86)	0.69 (0.56, 0.85)	1.21 (0.82, 1.72)	1.11 (0.74, 1.61)	0.97 (0.73, 1.27)	0.92 (0.68, 1.23)	0.87 (0.68, 1.10)	0.75 (0.58, 0.97)	0.80 (0.41, 1.42)	0.76 (0.38, 1.37)
	Black	0.66 (0.60, 0.71)	0.66 (0.61, 0.72)	0.68 (0.57, 0.81)	0.69 (0.57, 0.83)	0.64 (0.59, 0.71)	0.66 (0.59, 0.72)	0.58 (0.53, 0.64)	0.59 (0.53, 0.65)	0.67 (0.48, 0.92)	0.70 (0.50, 0.97)
	Native Hawaiian	0.80 (0.37, 1.51)	0.82 (0.37, 1.60)	1.58 (0.25, 5.67)	2.14 (0.33, 8.08)	0.29 (0.05, 0.94)	0.33 (0.05, 1.13)	0.86 (0.26, 2.15)	1.04 (0.31, 2.64)	2.23 (0.12, 12.62)	2.79 (0.15, 16.16)
	Hispanic	0.50 (0.46, 0.55)	0.60 (0.55, 0.66)	0.51 (0.39, 0.65)	0.58 (0.44, 0.75)	0.68 (0.61, 0.77)	0.78 (0.69, 0.89)	0.67 (0.59, 0.76)	0.76 (0.66, 0.86)	0.48 (0.27, 0.79)	0.58 (0.32, 0.96)
	Other	0.87 (0.64, 1.16)	0.96 (0.69, 1.31)	0.94 (0.39, 1.91)	1.02 (0.41, 2.20)	0.95 (0.66, 1.32)	1.29 (0.89, 1.82)	0.65 (0.42, 0.97)	0.84 (0.54, 1.27)	0.00 (0.00, 0.22)	0.00 (0.00, 0.23)
	Unknown	0.69 (0.60, 0.79)	0.77 (0.66, 0.89)	0.65 (0.47, 0.88)	0.64 (0.45, 0.88)	0.78 (0.66, 0.91)	0.84 (0.71, 0.99)	0.80 (0.68, 0.93)	0.88 (0.74, 1.03)	0.94 (0.60, 1.40)	1.09 (0.69, 1.65)
ICU admission		3.34 (3.01, 3.71)	2.64 (2.36, 2.96)	3.73 (2.84, 4.84)	2.66 (1.97, 3.55)	4.17 (3.74, 4.65)	3.07 (2.73, 3.46)	3.84 (3.39, 4.35)	2.95 (2.57, 3.37)	3.77 (2.64, 5.27)	2.97 (2.03, 4.27)
Cardiac disease		1.88 (1.77, 2.00)	1.47 (1.37, 1.57)	1.99 (1.72, 2.29)	1.49 (1.26, 1.76)	1.66 (1.56, 1.77)	1.32 (1.23, 1.42)	1.42 (1.32, 1.52)	1.29 (1.19, 1.39)	1.56 (1.27, 1.93)	1.46 (1.15, 1.84)
Diabetes M		1.38 (1.30, 1.46)	1.27 (1.19, 1.36)	1.62 (1.40, 1.86)	1.37 (1.17, 1.60)	1.62 (1.52, 1.72)	1.39 (1.30, 1.49)	1.33 (1.24, 1.43)	1.26 (1.17, 1.36)	1.17 (0.94, 1.45)	1.10 (0.87, 1.38)
Liver disease		1.19 (1.08, 1.31)	0.75 (0.67, 0.83)	1.55 (1.26, 1.89)	0.93 (0.74, 1.16)	1.40 (1.28, 1.54)	0.94 (0.85, 1.04)	1.23 (1.12, 1.35)	0.88 (0.80, 0.97)	1.37 (1.04, 1.77)	1.02 (0.76, 1.35)
Resp. disease		1.37 (1.27, 1.47)	1.12 (1.03, 1.22)	1.38 (1.16, 1.65)	1.13 (0.92, 1.37)	1.34 (1.25, 1.45)	1.12 (1.03, 1.22)	1.05 (0.97, 1.14)	0.94 (0.86, 1.03)	0.94 (0.73, 1.19)	0.84 (0.64, 1.08)
BMI≥30		0.85 (0.80, 0.91)	0.76 (0.71, 0.81)	0.98 (0.86, 1.13)	0.89 (0.76, 1.04)	1.21 (1.14, 1.29)	1.00 (0.93, 1.07)	0.95 (0.88, 1.02)	0.83 (0.77, 0.90)	0.87 (0.71, 1.08)	0.77 (0.61, 0.97)
Days in hospital		1.05 (1.05, 1.05)	1.04 (1.03, 1.04)	1.07 (1.06, 1.07)	1.05 (1.05, 1.06)	1.05 (1.05, 1.06)	1.04 (1.04, 1.05)	1.05 (1.04, 1.05)	1.03 (1.03, 1.04)	1.05 (1.04, 1.06)	1.04 (1.02, 1.05)
Treatment		1.36 (1.28, 1.44)	1.11 (1.04, 1.19)	1.08 (0.94, 1.24)	0.90 (0.77, 1.04)	1.45 (1.36, 1.54)	1.14 (1.07, 1.22)	1.60 (1.50, 1.72)	1.33 (1.23, 1.43)	1.34 (1.09, 1.65)	1.16 (0.93, 1.44)
AST^1^		1.04 (1.03, 1.04)	–	1.04 (1.03, 1.05)	–	1.03 (1.03, 1.04)	–	1.04 (1.03, 1.04)	–	1.04 (1.03, 1.05)	--
ALT^1^		1.00 (1.00, 1.01)	–	1.02 (1.01, 1.03)	–	1.01 (1.00, 1.01)	–	1.02 (1.02, 1.03)	–	1.03 (1.02, 1.04)	--
PLT^1^		0.97 (0.97, 0.98)	–	0.98 (0.97, 0.98)	–	0.97 (0.97, 0.98)	–	0.98 (0.98, 0.98)	–	0.98 (0.97, 0.99)	--
FIB-4		1.25 (1.24, 1.26)	1.22 (1.21, 1.23)	1.25 (1.22, 1.28)	1.21 (1.18, 1.24)	1.22 (1.21, 1.24)	1.20 (1.19, 1.21)	1.20 (1.19, 1.22)	1.19 (1.18, 1.20)	1.20 (1.16, 1.23)	1.18 (1.14, 1.21)
FIB-4 > 2.67		3.75 (3.53, 3.99)	–	3.59 (3.11, 4.14)	–	3.44 (3.23, 3.68)	–	3.55 (3.31, 3.82)	–	2.70 (2.18, 3.33)	–
FIB-4 > 3.04		3.87 (3.64, 4.11)	–	3.63 (3.15, 4.18)	–	3.44 (3.23, 3.66)	–	3.51 (3.27, 3.76)	--–	2.70 (2.19, 3.33)	–
FIB-4 > 3.25		3.79 (3.57, 4.02)	–	3.69 (3.21, 4.25)	–	3.38 (3.18, 3.60)	–	3.42 (3.19, 3.67)	–	2.84 (2.30, 3.51)	–

AST = alanine aminotransferase; BMI = body mass index; FIB-4 = fibrosis-4; ICU = intensive care unit; MR = multiple logistic regression; PLT = platelets; SR = simple logistic regression.

## Discussion

SARS-CoV-2 has evolved, with recent variants showing lower hospitalization rates [4]. With the earlier variants, several studies showed that FIB-4 had good diagnostic performance [1,7,9–11,18–20]. Although COVID-19 variants are continuously evolving over time and vaccination efforts have reduced the requirement of MV, it is useful to understand risk factors and identify high-risk patients on initial presentation. This analysis utilized a large sample size of patients, allowing us to study a large diverse population of hospitalized COVID-19 patients over time. Our analysis showed that hospitalized patients with COVID-19 and increased FIB-4 at any threshold (>2.67, >3.04, and>3.25) were 1.8 times more likely to require MV across all variants. The adjusted OR of MV use also increased when FIB-4 increased by one unit. In the adjusted model, for a FIB-4 index>3.04, all waves exhibited an increased OR of requiring MV. The multivariable model adjusted for diabetes, comorbid cardiac, liver, respiratory disease, and obesity and still showed an increased OR. This allows FIB-4 to further discriminate patients with multiple comorbidities that may require MV vs. patients with multiple comorbidities that are less likely to require MV.

In comparison with previous literature, which largely studied patients with the initial variant, Younossi *et al.* also showed that in hospitalized patients with NAFLD, a FIB-4 > 2.91 had a 26.5% rate of MV use vs. 12.8% in FIB-4 levels between 1.16 and 2.91 (*p* < 0.0001) during the initial variant [10]. Similar to our study, Samaniego *et al.* showed that a FIB-4 > 2.67 independently increased risk of MV by multivariate analysis, differing by a higher OR of 3.41 [1.30–8.92], compared to our OR 1.88 [1.77, 1.99] for the same variant (Table S3) [18]. Bucci *et al*. showed that a FIB-4 > 2.76 during the initial variant was associated with MV with a HR of 2.07 ([1.03–4.19], *p* = 0.043), which is in line with our study, albeit at a higher numeric value [19]. Similarly, Park *et al.* studied a higher FIB-4 threshold of≥4.95 and showed a HR of 2.784 ([1.691–4.585], *p* < 0.001) [20]. For AST levels, Samaniego *et al.* did not find a significant increase in odds of MV use with univariate analysis and multivariate analysis [18], while our study showed that a 10-unit increase in AST was significantly associated with MV at a univariable level, across all COVID-19 waves with an OR around 1.04 [1.03, 1.05] for each wave. ALT was also significantly associated with MV at a univariable level in our study across all waves, but did not show any significance in the study by Samaniego *et al*. [18] or Park *et al*. [20]

Several complex models have been developed to predict the mortality of hospitalized patients with COVID-19, however, have been challenging for general applicability to routine clinical care due to the use of non-routine laboratory values and the requirement of subjective symptoms in the calculations [12–14]. Also, few have focused on the comparison to need for MV, which can help predict patients at high risk of decompensation and is often a precursor to mortality. These scores include the 4C mortality score, BAS^2^IC score, and the red cell distribution width (RDW). The 4C score utilizes eight variables including c-reactive protein and level of consciousness and showed an overall high AUC of 0.79 [0.78, 0.79] for mortality and a high NPV at 99% [12]. The BAS^2^IC score included six components, which included dyspnea, neutrophil count, lymphocyte count, and c-reactive protein, and found a NPV of 87% [13]. One simple test, increased RDW, was shown to be associated with increased mortality from COVID-19, but did not show an association with MV [1,14].

SARS-CoV-2 has shown a hepatocellular pattern of acute liver injury with AST greater than ALT, thus myocyte or muscular injury may also contribute [21–23]. There are, however, case reports of new-onset dermatomyositis post-SARS-CoV-2 infection or vaccination suggesting a potential association of the virus with direct muscular injury [24]. Our studies are in line with prior studies that did not show any significant association between MV and platelets [23].

The AUROC curves between the continuous FIB-4 covariate and MV use per COVID-19 variant wave showed a test with a high sensitivity and excellent accuracy for predictability of need for MV in hospitalized patients by FIB-4. The NPV showed utility—allowing providers to quickly triage patients who may have a lower risk of impending respiratory failure vs. those patients with a high FIB-4 index, who have a higher risk of respiratory failure requiring MV. The PPV was low, showing the greater utility of FIB-4 as a negative predictor, which also relies on the prevalence of the disease in the population.

Our study showed that using SR and MR modeling for FIB-4 as a continuous variable (Table 3), there was an increased OR of 1.21 [1.20, 1.21] for 30-day mortality throughout all waves without significant variability between variants. Other studies have assessed odds and hazard ratios of mortality using varying FIB-4 parameters and data analysis methods. Li *et al.* utilized multivariate logistic regression and proportional hazards modeling and showed that a one-unit increase in FIB-4 was associated with an increase in odds of death at 1.79 ([1.36, 2.35], *p* < .0001) [9], which were lower than our findings during the initial variant in 2020 (OR = 1.22). Park *et al.* utilized the Kaplan Meier for overall survival analysis and describe adjusted HR of 3.02 [1.84–4.96] for mortality utilizing a higher threshold at FIB-4 ≥ 4.95 [20], whereas the highest threshold in our study was 3.25. Park *et al.* found FIB-4 as an independent risk factor for mortality [20]. Our findings for mortality are lower than previous studies by Sterling *et al.*, which utilized the Student’s *T* test and showed that a FIB-4 > 2.67 had an increased 30-day mortality with an OR of 8.4 (2.23–31) [11]. Bucci *et al*. utilized COX regression analysis and showed an adjusted HR of 1.72 ([1.14–2.59], *p* = 0.010) for a FIB-4 > 3.25, also showing a higher mortality with increased FIB-4 levels [19]. In line with prior research, our findings showed consistent positive association between the FIB-4 index and risk of mortality among hospitalized COVID-19 patients among all SARS-CoV-2 variants.

### Limitations

For the components of the FIB-4 index calculation, we did not exclude patients previously diagnosed with thrombocytopenia, myositis and myopathies, rhabdomyolysis, elevated creatine kinase levels, or another attributable medical cause of acute or chronic elevations in AST, which can affect the calculation of the FIB-4 index [23]. Our study had a mean age of 56–62 and included ages 18–90 years old; thus, results cannot be generalized to children under 18. Both older and younger ages can cause over- or under-estimation of fibrosis level by FIB-4 index. N3C is a consortium database sourced from many institutions with multiple data models that have been harmonized into the OMOP common data model through a process that could potentially introduce error including missing data points. Additionally, not all patients had the laboratory values needed to calculate the FIB-4 index, thus missing data and excluding these patients from the analysis may have impacted our results. Other ways to validate these findings include a future study conducting a large prospective cohort study, allowing us to follow patients over time.

Although we studied use of MV with patient’s receiving treatment for COVID-19, the specific treatment course or type and its effect on outcomes was not analyzed and the focus of this study. Our data show that, during the initial, Delta, and Omicron-initial waves, patients treated for COVID-19 had higher odds of requiring MV; there was no difference for Omicron-subsequent. This suggests that these patients may have required treatment due to a more severe hospital course and thus likely also had a higher 30-day mortality. Future studies should address this phenomenon and study the treatments and their effects on patient outcomes closely. This study utilized data from patients who required mechanical ventilation thus it utilizes ICU-level data. A study of floor-status patients may be helpful for a different component of a COVID-19 study but it was not available in this data set. As MV requires ICU-level care, we focused our analysis on that population. We acknowledge that patients who are initially admitted to the “floor” may deteriorate and require transfer to the ICU. In that case, they would be included in our analysis of ICU cases.

## Conclusion

The FIB-4 index was consistently and positively associated with increased utilization of MV and increased risk of mortality among hospitalized COVID-19 patients. This effect was consistent across all COVID-19 variant waves and in both adjusted and unadjusted models. The analysis suggests that the FIB-4 index continues to predict respiratory failure requiring MV support from COVID-19 across all VOC including Alpha, Delta, and Omicron. This provides a simple tool based on routinely available tests for risk-stratification using baseline admission laboratory values for front-line healthcare professionals for future variants.

## Supporting information

Parajuli et al. supplementary material Parajuli et al. supplementary material

## Supplementary material

The supplementary material for this article can be found at https://doi.org/10.1017/cts.2023.594.
