# Supplementary material for: Fibrosis-4 (FIB-4) index as a predictor for mechanical ventilation and 30-day mortality across COVID-19 variants
Source: J Clin Transl Sci. 2023 Jul 17;7(1):e213. doi: 10.1017/cts.2023.594 (PMC10643913; doi:10.1017/cts.2023.594)
Supplement: Parajuli et al. supplementary material [file S2059866123005940sup001.docx]

# Supplemental

#### **Table S1:** Date ranges of COVID-19 variants.

| Variant | Start Date | End Date |
| --- | --- | --- |
| Initial | April 27, 2020 | November 23, 2020 |
| Alpha | March 29, 2021 | May 24, 2021 |
| Delta | August 2, 2021 | December 6, 2021 |
| Initial Omicron | December 20, 2021 | March 14, 2022 |
| Subsequent Omicron | March 28, 2022 | June 25, 2022 |

#### **Table S2:** Patient Demographics and Clinical Characteristics by COVID Variants.

| Characteristic | Value | Initial (n) | Alpha (n) | Delta (n) | Omicron-initial (n) | Omicron- subsequent (n) |
| --- | --- | --- | --- | --- | --- | --- |
| Sex | Male | 25422 (53.05 %) | 6010 (49.23 %) | 19859 (52.00 %) | 17888 (51.30 %) | 3431 (49.62 %) |
|  | Female | 22497 (46.95 %) | 6197 (50.77 %) | 18328 (48.00 %) | 16983 (48.70 %) | 3484 (50.38 %) |
| Cardiac Disease | Yes | 14369 (29.99 %) | 3192 (26.15 %) | 11565 (30.29 %) | 14653 (42.02 %) | 3304 (47.78 %) |
|  | No | 33550 (70.01 %) | 9015 (73.85 %) | 26622 (69.71 %) | 20218 (57.98 %) | 3611 (52.22 %) |
| Diabetes | Yes | 16712 (34.88 %) | 3702 (30.33 %) | 12113 (31.72 %) | 12861 (36.88 %) | 2373 (34.32 %) |
|  | No | 31207 (65.12 %) | 8505 (69.67 %) | 26074 (68.28 %) | 22010 (63.12 %) | 4542 (65.68 %) |
| Liver Disease | Yes | 4484 (9.36 %) | 1188 (9.73 %) | 4112 (10.77 %) | 5255 (15.07 %) | 1043 (15.08 %) |
|  | No | 43435 (90.64 %) | 11019 (90.27 %) | 34075 (89.23 %) | 29616 (84.93 %) | 5872 (84.92 %) |
| Respiratory Disease | Yes | 7534 (15.72 %) | 1831 (15.00 %) | 6652 (17.42 %) | 8300 (23.80 %) | 1764 (25.51 %) |
|  | No | 40385 (84.28 %) | 10376 (85.00 %) | 31535 (82.58 %) | 26571 (76.20 %) | 5151 (74.49 %) |
| Ventilator | Yes | 4759 (9.93 %) | 975 (7.99 %) | 4487 (11.75 %) | 3067 (8.80 %) | 395 (5.71 %) |
|  | No | 43160 (90.07 %) | 11232 (92.01 %) | 33700 (88.25 %) | 31804 (91.20 %) | 6520 (94.29 %) |
| Death in Hospital | Yes | 4327 (9.03 %) | 798 (6.54 %) | 4296 (11.25 %) | 2911 (8.35 %) | 267 (3.86 %) |
|  | No | 43592 (90.97 %) | 11409 (93.46 %) | 33891 (88.75 %) | 31960 (91.65 %) | 6648 (96.14 %) |
| Treatment | Yes | 14950 (31.20 %) | 5413 (44.34 %) | 19886 (52.08 %) | 14446 (41.43 %) | 3034 (43.88 %) |
|  | No | 32969 (68.80 %) | 6794 (55.66 %) | 18301 (47.92 %) | 20425 (58.57 %) | 3881 (56.12 %) |
| Race/Ethnicity | White, NH | 24035 (50.16 %) | 6186 (50.68 %) | 25702 (67.31 %) | 21108 (60.53 %) | 4610 (66.67 %) |
|  | Black/AA | 9568 (19.97 %) | 3172 (25.99 %) | 6463 (16.92 %) | 7407 (21.24 %) | 1072 (15.50 %) |
|  | Hispanic or Latino | 9791 (20.43 %) | 1555 (12.74 %) | 3383 (8.86 %) | 3351 (9.61 %) | 504 (7.29 %) |
|  | Unknown | 2563 (5.35 %) | 810 (6.64 %) | 1718 (4.50 %) | 1850 (5.31 %) | 441 (6.38 %) |
|  | Asian, NH | 1253 (2.61 %) | 345 (2.83 %) | 446 (1.17 %) | 715 (2.05 %) | 225 (3.25 %) |
|  | Other, NH | 440 (0.92 %) | 89 (0.73 %) | 294 (0.77 %) | 300 (0.86 %) | 36 (0.52 %) |
|  | American Indian or Alaska Native, NH | 182 (0.38 %) | <35 (* %) | 135 (0.35 %) | 101 (0.29 %) | <20 (* %) |
|  | Native Hawaiian, PSL | 87 (0.18 %) | <20 (* %) | 46 (0.12 %) | 39 (0.11 %) | <20 (* %) |
| Obesity | Yes | 22525 (47.01 %) | 6266 (51.33 %) | 17546 (45.95 %) | 15783 (45.26 %) | 3066 (44.34 %) |
|  | No | 25394 (52.99 %) | 5941 (48.67 %) | 20641 (54.05 %) | 19088 (54.74 %) | 3849 (55.66 %) |
| FIB-4 | Mean (SD) | 2.70(2.34) | 2.58(2.24) | 2.94(2.54) | 2.76(2.62) | 2.65(2.52) |
| Age (years) | Mean (SD) | 60.39(17.08) | 56.52(16.76) | 58.17(16.99) | 60.14(17.91) | 62.93(18.91) |
| AST (U/L) | Mean (SD) | 48.73(54.50) | 51.58(55.15) | 56.67(61.54) | 48.51(64.94) | 41.52(59.05) |
| ALT (U/L) | Mean (SD) | 39.65(49.46) | 42.34(52.80) | 43.48(54.60) | 37.01(56.01) | 34.00(60.39) |
| PLT (10^9^/L) | Mean (SD) | 218.65(93.09) | 218.22(93.03) | 216.61(96.58) | 224.62(101.26) | 220.44(97.80) |

*Abbreviations: Non-Hispanic (NH), African American (AA), Pacific Islander (PSI), Body Mass Index (BMI), Alanine aminotransferase (AST), Aspartate aminotransferase (AST), Platelets (PLT), Fibrosis-4 (FIB-4)*

** Proportion too small to quantitatively report.*

**Table S3.** Odds Ratios of Mechanical Ventilation Using Simple vs. Multiple Logistic Regression Models for the Initial Variants and Other variants.

| Wave | | Initial | | Other (includes alpha, delta, omicron) | |
| --- | --- | --- | --- | --- | --- |
|  | | SLR | MLR | SLR | MLR |
| Variables | | OR (95% CI) | OR (95% CI) | OR (95% CI) | OR (95% CI) |
| Intercept |  | -- | 0.01 (0.01, 0.01) | -- | 0.01 (0.01, 0.02) |
| Female |  | 0.66 (0.62, 0.71) | 0.76 (0.71, 0.82) | 0.64 (0.61, 0.67) | 0.71 (0.68, 0.75) |
| Age* |  | 1.04 (1.02, 1.05) | -- | 1.00 (0.98, 1.01) | -- |
| Race | Native American | 2.58 (1.79, 3.73) | 1.91 (1.23, 2.95) | 1.99 (1.44, 2.74) | 1.32 (0.89, 1.95) |
|  | Asian | 1.31 (1.09, 1.57) | 1.30 (1.05, 1.61) | 1.72 (1.56, 1.90) | 1.58 (1.40, 1.77) |
|  | Black | 1.26 (1.16, 1.36) | 1.16 (1.06, 1.27) | 1.18 (1.12, 1.25) | 1.04 (0.97, 1.11) |
|  | Native Hawaiian | 1.52 (0.80, 2.86) | 0.88 (0.41, 1.89) | 1.65 (1.00, 2.72) | 1.71 (0.97, 3.02) |
|  | Hispanic | 1.37 (1.27, 1.48) | 1.26 (1.15, 1.39) | 1.60 (1.51, 1.69) | 1.44 (1.35, 1.55) |
|  | Other | 0.88 (0.62, 1.25) | 1.10 (0.74, 1.63) | 1.02 (0.78, 1.33) | 1.04 (0.76, 1.42) |
|  | Unknown | 1.31 (1.15, 1.49) | 1.45 (1.24, 1.69) | 1.35 (1.24, 1.45) | 1.20 (1.09, 1.31) |
| ICU admission |  | 8.03 (7.31, 8.83) | 5.00 (4.48, 5.58) | 7.25 (6.71, 7.84) | 5.32 (4.85, 5.82) |
| Cardiac Disease |  | 1.02 (0.95, 1.08) | 0.77 (0.71, 0.84) | 0.81 (0.77, 0.85) | 0.70 (0.66, 0.74) |
| Diabetes M |  | 1.86 (1.75, 1.97) | 1.45 (1.35, 1.56) | 1.60 (1.54, 1.67) | 1.35 (1.28, 1.42) |
| Liver Disease |  | 2.05 (1.89, 2.23) | 1.53 (1.38, 1.70) | 1.96 (1.85, 2.07) | 1.62 (1.51, 1.74) |
| Respiratory Disease |  | 0.95 (0.87, 1.03) | 0.96 (0.87, 1.07) | 0.78 (0.73, 0.82) | 0.85 (0.79, 0.92) |
| BMI≥30 |  | 1.62 (1.52, 1.72) | 1.32 (1.22, 1.42) | 1.53 (1.47, 1.59) | 1.43 (1.36, 1.50) |
| Days in Hospital |  | 1.10 (1.09, 1.10) | 1.09 (1.08, 1.09) | 1.10 (1.10, 1.10) | 1.09 (1.09, 1.10) |
| Treatment |  | 2.75 (2.58, 2.92) | 2.19 (2.04, 2.35) | 1.52 (1.46, 1.59) | 1.33 (1.27, 1.40) |
| AST* |  | 1.04 (1.04, 1.05) | -- | 1.04 (1.04, 1.04) | -- |
| ALT* |  | 1.02 (1.02, 1.03) | -- | 1.03 (1.03, 1.03) | -- |
| PLT* |  | 1.00 (0.99, 1.00) | -- | 0.99 (0.99, 1.00) | -- |
| FIB-4 |  | 1.13 (1.12, 1.14) | 1.10 (1.09, 1.12) | 1.11 (1.10, 1.11) | 1.09 (1.08, 1.10) |
| FIB-4>2.67 |  | 1.88 (1.77, 1.99) | -- | 1.71 (1.64, 1.78) | -- |
| FIB-4>3.04 |  | 1.91 (1.80, 2.03) | -- | 1.73 (1.66, 1.81) | -- |
| FIB-4>3.25 |  | 1.86 (1.75, 1.98) | -- | 1.76 (1.69, 1.84) | -- |

*Abbreviations: Simple Logistic Regression (SLR), Multiple Logistic Regression (MLR), Intensive Care Unit (ICU),*

*Body Mass Index (BMI), Alanine aminotransferase (AST), Aspartate aminotransferase (AST), Platelets (PLT), Fibrosis-4 (FIB-4)*

**Odds ratios for 10-unit difference*

All COVID+ Patients

6,119,102

Initial Wave

47,919

Valid Death Dates and Sex

232,364

Alpha Wave

12,207

Delta Wave

38,187

Omicron-initial Wave

34,871

Omicron-subsequent Wave

6,915

Between Waves

92,265

Ages 18-90

5,060,132

Valid FIB-4 Labs

232,524

#### **Figure S1.** Breakdown of the sample size of COVID-19 positive patients by age, valid FIB-4 levels, and valid death dates.


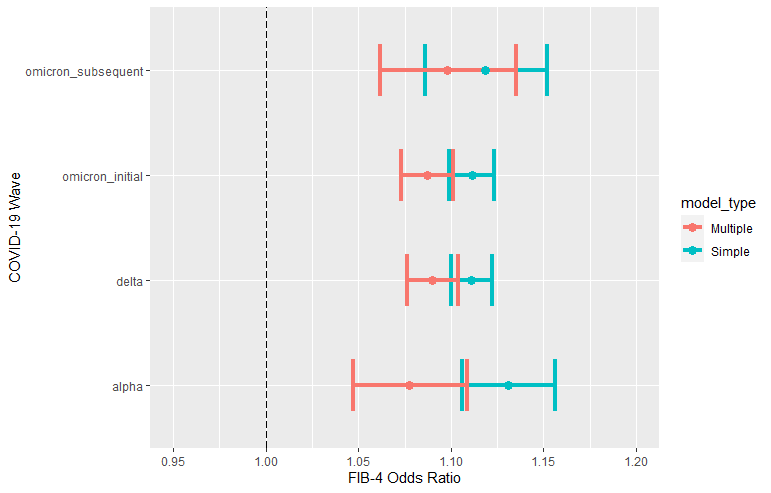


#### **Figure S2.** Odds Ratios for Mechanical Ventilation for Fibrosis-4 (FIB-4) by COVID-19 Variant Using Simple vs. Multiple Logistic Regression Modeling (FIB-4 as a continuous variable).


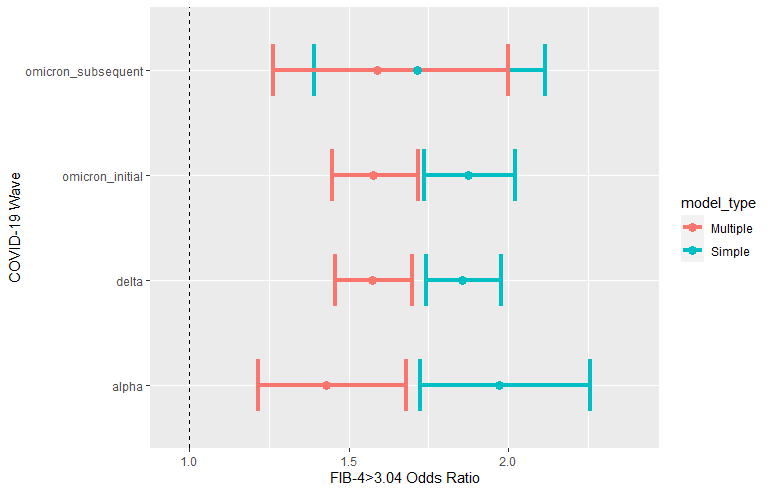


**Figure S3.** Odds Ratios for Mechanical Ventilation for Fibrosis-4 (FIB-4) > 3.04 by COVID-19 Variant Using Simple vs. Multiple Logistic Regression Modeling.


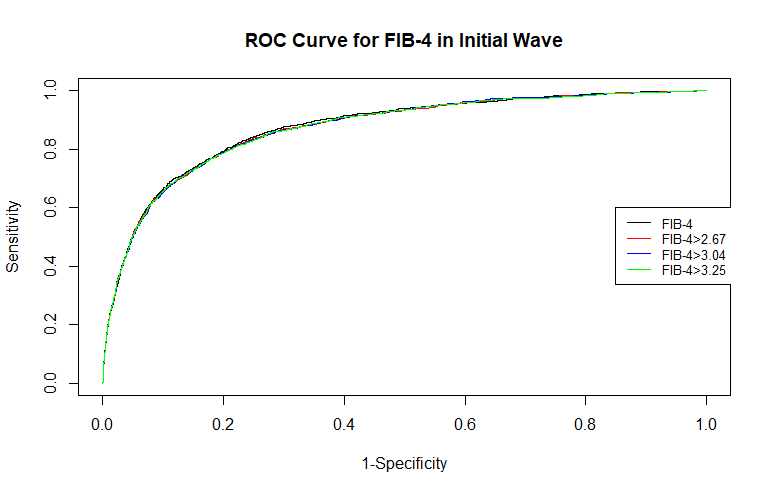


**Figure S4.** Receiver Operating Characteristic (ROC) Curve by FIB-4 thresholds for the Initial COVID-19 Variant.


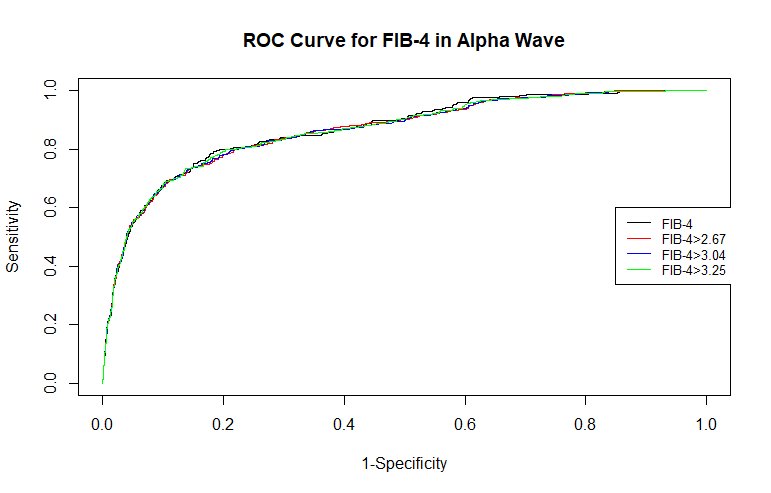


**Figure S5.** Receiver Operating Characteristic (ROC) Curve by FIB-4 thresholds for the Alpha COVID-19 Variant.


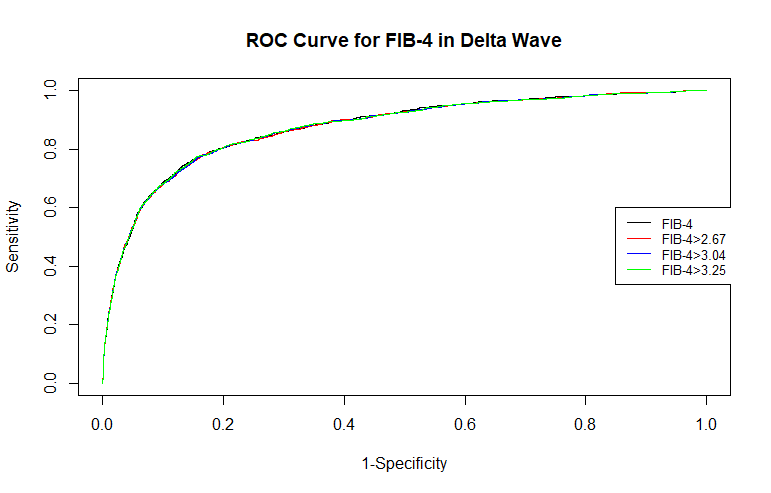
**Figure S6.** Receiver Operating Characteristic (ROC) Curve by FIB-4 thresholds for the Delta COVID-19 Variant.


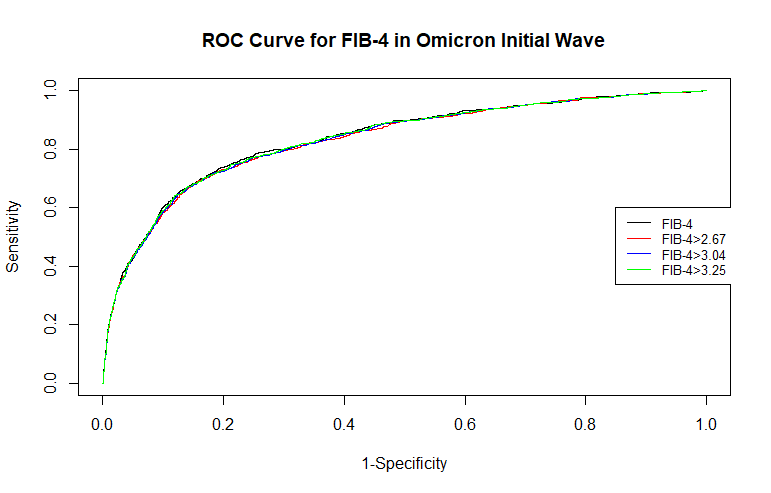
**Figure S7.** Receiver Operating Characteristic (ROC) Curve by FIB-4 thresholds for the Omicron-Initial COVID-19 Variant.


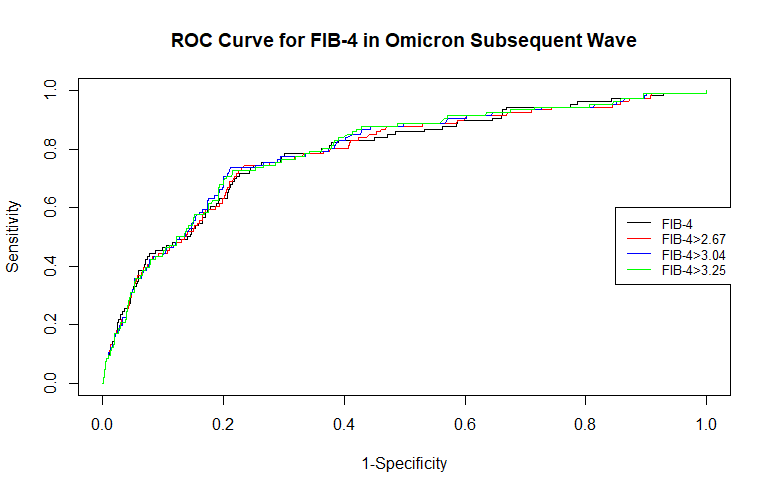
**Figure S8.** Receiver Operating Characteristic (ROC) Curve by FIB-4 thresholds for the Omicron-Subsequent COVID-19 Variant.
